# Aß Pathology and Neuron–Glia Interactions: A Synaptocentric View

**DOI:** 10.1007/s11064-022-03699-6

**Published:** 2022-08-17

**Authors:** Christiaan F. M. Huffels, Jinte Middeldorp, Elly M. Hol

**Affiliations:** 1 Department of Translational Neuroscience, University Medical Center Utrecht Brain Center, Utrecht University, Utrecht, The Netherlands; 2 Department of Neurobiology & Aging, Biomedical Primate Research Centre, Rijswijk, The Netherlands

**Keywords:** Alzheimer’s disease, Amyloid-ß, Synapse, Astrocyte, Microglia, Glia

## Abstract

Alzheimer’s disease (AD) causes the majority of dementia cases worldwide. Early pathological hallmarks include the accumulation of amyloid-ß (Aß) and activation of both astrocytes and microglia. Neurons form the building blocks of the central nervous system, and astrocytes and microglia provide essential input for its healthy functioning. Their function integrates at the level of the synapse, which is therefore sometimes referred to as the “quad-partite synapse”. Increasing evidence puts AD forward as a disease of the synapse, where pre- and postsynaptic processes, as well as astrocyte and microglia functioning progressively deteriorate. Here, we aim to review the current knowledge on how Aß accumulation functionally affects the individual components of the quad-partite synapse. We highlight a selection of processes that are essential to the healthy functioning of the neuronal synapse, including presynaptic neurotransmitter release and postsynaptic receptor functioning. We further discuss how Aß affects the astrocyte’s capacity to recycle neurotransmitters, release gliotransmitters, and maintain ion homeostasis. We additionally review literature on how Aß changes the immunoprotective function of microglia during AD progression and conclude by summarizing our main findings and highlighting the challenges in current studies, as well as the need for further research.

## Introduction

**Fig. 1:**
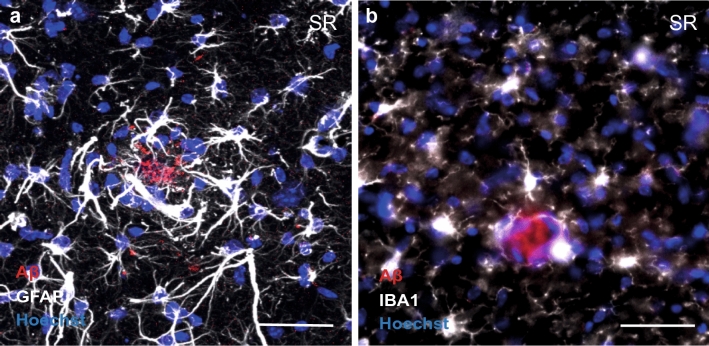
Pathological hallmarks of AD include the presence of Aβ plaques, and reactivity of astrocytes and microglia. **a** An Aβ plaque (red) surrounded by reactive astrocytes in the stratum radiatum (SR) of a 9-month-old APPswe/PSEN1dE9 mouse. Activated astrocytes (white) undergo clear cytoskeletal changes in response to Aβ pathology. **b** An Aβ plaque (red) surrounded by activated microglia (white) in the SR of a 9-month-old APPswe/PSEN1dE9 mouse. Microglia respond to Aβ pathology and are actively involved in clearing Aβ due to their phagocytotic capacity. Hoechst nuclei staining is indicated in blue. Scale bars: 50 µm

AD constitutes the largest known form of dementia, with current estimates ranging from 25 to 50 million people suffering from AD worldwide [1]. AD patients either suffer from familial AD or develop AD on a sporadic basis [2]. The familial form is caused by specific missense mutations in the amyloid precursor protein (APP) and presenilin 1 and 2 (PSEN1, PSEN2), and symptoms usually develop between the age of 30 and 50 [3]. Sporadic late-onset AD, however, is expected to develop due to a complex interplay between genetic and environmental factors [4]. Biologically, AD is a neurodegenerative disease that is characterized by pathological hallmarks, including the accumulation of amyloid-ß (Aß) peptides and phosphorylation of tau protein, resulting in the presence of Aß plaques (Fig. 1a, b) and neurofibrillary tangles (NFTs), respectively [5]. NFTs are specific for late-stage disease progression [6, 7]. The initial phase of AD development is typically characterized by the accumulation of Aß peptides [7], which are derivatives of cleavage of the APP, which is a transmembrane protein abundantly expressed by neurons, particularly at the synapse [8]. The APP is considered important for synaptic transmission and its expression is strictly regulated. Up- or downregulation of the APP negatively impacts synaptic plasticity and cognitive performance, as indicated by reduced performance on behavioral paradigms and impaired levels of long-term potentiation [9–12]. Regulatory processes include cleavage and breakdown of the APP, which, in the case of AD, deteriorate and initiate a cascade of events that ultimately leads to cognitive decline. In AD, excessive APP cleavage results in a relative shift of Aß peptides, increasing the Aß_42_/Aß_40_ ratio [13]. These longer Aß peptides (e.g. Aß_42_) have an increased aggregation capacity and progressively form Aß oligomers [14]. Currently, especially these Aß oligomers are considered toxic for neurotransmission. As the APP is abundantly expressed at the synapse and Aß accumulation is apparent at the start of AD pathogenesis, it has been suggested that AD is a synaptopathy [15], in which pre- and postsynaptic processes progressively deteriorate.

In addition to its direct effect on neuronal function, Aß accumulation affects the function of glial cells. Glial cells include a range of cell types, including astrocytes and microglia [16]. They act as important modulators of synaptic transmission and do so by closely interacting with the pre- and postsynapse. Astrocyte processes encapsulate the synaptic cleft and ensure recycling of released neurotransmitters, release co-factors important for physiological neuronal transmission, and maintain tissue ion homeostasis [17]. Astrocytes are connected via gap-junction-coupled networks that synchronize neuronal activity within brain regions and prevent focal epileptic seizures [17]. Microglia are the resident immune cells of the brain and constantly explore the environment for pathogens [16]. They phagocytose inactive synapses and release co-factors that are important for the induction and maintenance of synaptic plasticity [18–21]. Together, astrocytes and microglia are considered essential parts of the neuronal synapse. As such, the synaptic complex is sometimes referred to as the “quad-partite synapse” [22], consisting of the pre- and postsynapse, the astrocyte, and microglia.

The components of the quad-partite synapse have been studied extensively using many different approaches, ranging from post-mortem AD patient material, to in-vitro and in-vivo model systems. In-vivo model systems include AD mouse models, which often express a humanized chimeric form of the APP combined with the expression of a mutated form of PSEN, which is the active part of the gamma-secretase complex and actively cleaves the APP [5]. There are several AD mouse models available based on familial mutations in the APP, PSEN1, PSEN2, and risk genes APOE4 and TREM1. These models are characterized by the in-vivo accumulation of Aß peptides that develop into Aß oligomers and ultimately fibrillar plaque deposits. These models develop deficits across cognitive domains, including contextual and spatial memory, and impairments at the microcircuit level include activation of astrocytes and microglia near Aß plaques (Fig. 1a, b), neuronal hyperactivity, and impaired synaptic plasticity [23].

The need for an AD treatment and the awareness that Aß pathology plays a key role in AD progression, as well as the readily available AD mouse models, has triggered a growing interest in the effect of Aß accumulation on synaptic physiology. Here, we aimed to discuss the current view on how Aß pathology in AD affects the individual components of the quad-partite synapse. We first discuss the components of the quad-partite synapse separately and conclude by summarizing our main findings and highlighting the challenges in current research.

## Neurons

A healthy balance between presynaptic neurotransmitter release and postsynaptic activation of α-amino-3-hydroxy-5-methyl-4-isoxazolepropionic acid receptors (AMPARs) and N-methyl-d-aspartate receptors (NMDARs) is essential for physiological neurotransmission. Impaired neurotransmission is a key characteristic for AD progression, and loss of functional synapses is considered an important pathological correlate of AD severity [24, 25]. Aβ fulfils an important regulatory role in preserving synaptic transmission within its physiological limits. Experiments indicate that neuronal activity and Aβ expression maintain a tight balance, where increased neuronal activity induces Aβ production, which in turn suppresses synaptic transmission. Indeed, administration of Aβ in low concentrations improved the induction of synaptic plasticity in the hippocampal CA1, whereas high concentrations resulted in the opposite effect [26, 27]. As such, excessive accumulation of Aß acts as a synaptotoxin and can interfere with synapse function directly [28]. Aß demonstrates a high affinity with receptors and proteins expressed at the pre- and postsynaptic membrane and prevents their physiological functioning [29–31]. This disrupts regulatory processes that are essential for the induction and maintenance of synaptic plasticity, such as receptor exo- and endocytosis, receptor trafficking and mobilization, and receptor conductance and subunit composition [32–35]. As such, the direct administration of Aß has been shown to inhibit the induction of synaptic plasticity and alter the physiological properties of neurons [36]. Central to the adverse effect of Aß are changes in pre- and postsynaptic Ca^2+^ homeostasis [37], of which tight regulation is essential for the changes in gene expression to occur that ultimately induce synaptic plasticity. The various ways in which Aß affects Ca^2+^ homeostasis are biologically diverse and the most important ones will be discussed below.

### Presynaptic Activity

Presynaptic nerve terminals accommodate a large range of biological mechanisms, which include the transport of cargo towards the synapse, and processes related to neurotransmitter recycling and release. Exposure to Aß pathology is associated with aberrant presynaptic physiology. Studies indicate that exposure to Aβ pathology interferes with the presynaptic release of neurotransmitters in multiple systems, including glutamatergic, γ-aminobutyric acid (GABA)ergic, and serotoninergic circuitry [38].

**Fig. 2:**
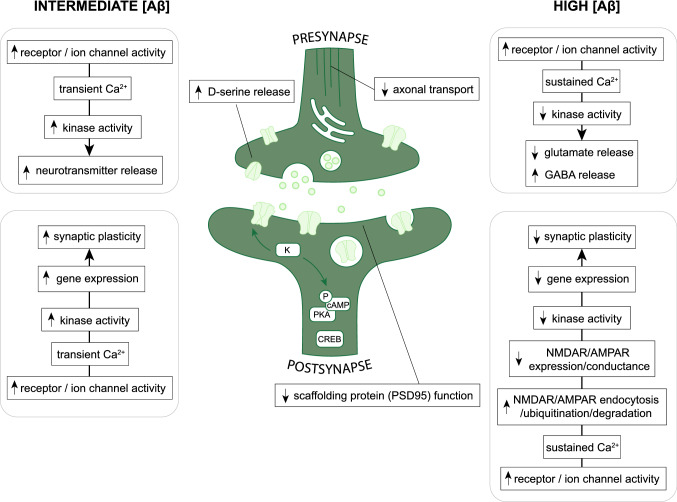
Aβ accumulation affects pre- and postsynaptic neurotransmission. Aβ stimulates presynaptic RyR, voltage-gated Ca^2+^ channel, and α7-nAChR activity. This increases the presynaptic [Ca^2+^]. Short-term fluctuations in the presynaptic [Ca^2+^] promote kinase (K) activity and stimulate neurotransmitter release. Long-term [Ca^2+^] increases result in presynaptic depression and a subsequent decrease in neurotransmitter (i.e., glutamate) release. More advanced stages of Aβ pathology are associated with enhanced GABA release and thus, an increase in tonic neuronal network inhibition. Aβ accumulation has furthermore been shown to affect axonal transport and presynaptic d-serine release. Postsynaptically, Aβ stimulates NMDAR and AMPAR subunit phosphorylation. This initially promotes NMDAR and AMPAR expression and conductance. Aβ additionally binds and activates postsynaptic α7-nAChRs. Subsequent increases in the postsynaptic [Ca^2+^] stimulate kinase activity and activate downstream pathways and gene expression important for synaptic plasticity induction and maintenance. Prolonged increases in the postsynaptic [Ca^2+^] stimulate endocytosis, ubiquitination, and degradation of NMDARs and AMPARs. The subsequent reduction in NMDAR and AMPAR expression and function results in postsynaptic depression and a reduction in gene transcription important for synaptic plasticity induction. Figure was created with the help of BioRender.com

The effect of Aβ on presynaptic physiology appears to be dose-dependent (Fig. 2). Low concentrations of extracellular Aβ increased presynaptic glutamate release without affecting postsynaptic activity [39–41], presumably by stimulating vesicle fusion with the presynaptic membrane. Aß additionally affects synaptic transmission via the increased release of co-factors important for postsynaptic receptor function. An important co-factor is D-serine, which binds to, and activates, postsynaptic NMDARs. Experiments indicate that Aβ administration enhances the extracellular presence of D-serine, possibly due to activation of presynaptic alanine-serine-cysteine transporter 1 (asc-1) [42, 43]. The enhanced release of presynaptic glutamate and D-serine in the presence of Aß augments its concentration in the synaptic cleft. As such, hyper-excitability and excitotoxicity are key characteristics of early AD development [44, 45].

High concentrations of Aβ reduce presynaptic glutamate release and promote excessive GABA release by interneurons in the hippocampal CA1 (Fig. 2) [40, 41, 46]. This is partially mediated by the effect of Aβ on acetylcholine circuitry, as Aβ interacts with cholinergic receptors in a dose-dependent manner [30, 31, 47, 48]. Acetylcholine as a neurotransmitter is essential for attention, learning and memory, and high concentrations of Aβ have been shown to interfere with α7-containing nicotinic acetylcholine receptor (α7-nAChR) activation in such a way that neurotransmitter release was reduced [40]. This dual effect of Aß pathology underscores the importance of maintaining healthy Aß concentrations and indicates that Aß accumulation induces a transition in which the neuronal network becomes increasingly inhibitory in nature with progressive Aß pathology.

Presynaptic physiology and mechanisms associated with neurotransmitter release all highly depend on the intracellular [Ca^2+^]. The presynaptic [Ca^2+^] results from intra- and extracellular Ca^2+^ sources. Studies indicate that Aß pathology affects the presynaptic [Ca^2+^] directly. For instance, Aß displays a high affinity to presynaptic voltage-gated Ca^2+^ channels, enhances their activity, and promotes the influx of Ca^2+^ [49]. An important intracellular Ca^2+^ source is the endoplasmatic reticulum (ER), which extends towards presynaptic axon terminals [50, 51]. The efflux of Ca^2+^ is predominantly provided by ryanodine receptors (RyR), which are abundantly expressed in the ER membrane [52]. The upregulation of RyR expression correlates with an increased presynaptic [Ca^2+^] and was associated with the impaired induction of synaptic plasticity in a mouse model for AD [53]. The same upregulation of RyR altered the paired-pulse facilitation in APP/PS1 mice, affecting neurotransmitter release [53–55]. The application of RyR inhibitors rescued this effect, illustrating the importance of intracellular Ca^2+^ sources and enhanced RyR activity in presynaptic pathology present in AD.

The Aß-induced increase in the intracellular [Ca^2+^] disturbs presynaptic mechanisms, including enzymatic phosphorylation, which ultimately affects axonal transport, neurotransmitter vesicle trafficking, recycling, and release, in a dual manner [29, 56, 57]. That is, Aß exposure results in the enhanced activity of presynaptic cyclin-dependent kinase 5, which reduces the neurotransmitter vesicle recycling pool and increases the number of resting neurotransmitter vesicles [56], thereby reducing presynaptic activity. Simultaneously, reports indicate that Aβ stimulates the phosphorylation of proteins in the SNARE complex, such as syntaxin 1, and competes with synaptobrevin and VAMP2 for binding with synaptophysin, which promotes presynaptic vesicle fusion [58–60]. This apparently conflicting effect of Aß on presynaptic activity likely results from a gradual increase in the presynaptic [Ca^2+^], where moderate increases initially stimulate biological pathways but prolonged exposure to Aß ultimately depresses presynaptic transmission. Nevertheless, how exactly Aß exerts its dual effect, what downstream pathways are involved, and how they are temporally regulated remains to be elucidated.

Overall, Aβ pathology affects presynaptic activity in many ways that ultimately converge into the altered release of neurotransmitters (Fig. 2). Initially, low concentrations of Aβ support synaptic transmission, but the excessive accumulation of Aβ in AD triggers the transition towards excitotoxicity and prolonged exposure to Aβ ultimately results in a network that is characterized by presynaptic depression.

### Postsynaptic AMPA Receptors

AMPARs are expressed at the postsynapse and their physiology is strictly regulated. Their activation facilitates depolarization of the postsynapse through the influx of Na^+^. AMPARs are heterotetramers that generally consist of two symmetric dimers containing one of four subunits; GluA1, GluA2, GluA3, and GluA4 [61]. The AMPAR subunit composition greatly affects its function and is regulated in a spatiotemporal manner [61, 62]. GluA4 heterodimers are exclusively expressed in the hippocampus during early development and are replaced by heterodimers containing GluA1, GluA2, and GluA3 subunits in adulthood [63]. The effect of early Aß pathology on AMPAR physiology appears to be subunit-specific. That is, exposure to Aß facilitates phosphorylation of the GluA1 subunit, which results in enhanced AMPAR exocytosis and increased AMPAR expression at the postsynaptic membrane [64]. It additionally raises AMPAR channel conductance and immobilizes AMPARs to ensure stable incorporation of GluA1-containing AMPARs into the postsynaptic density (PSD) [65, 66]. The Aß-induced increase in GluA1-containing AMPARs goes at the expense of GluA2-containing AMPAR expression [67, 68]. The absence of the GluA2 subunit renders AMPAR permeable to Ca^2+^, increasing the likelihood of local postsynaptic increases in the [Ca^2+^] as a result of Aß pathology [69]. Interestingly, it appears that the expression of the GluA3 AMPAR subunit is essential for Aß to exert its pathological effect at the initial stages of AD progression. That is, hippocampal neurons in APP/PS1 mice showed a reduced AMPAR-mediated response when exposed to Aß, whereas GluA3-deficient APP/PS1 mice did not [70].

Prolonged exposure to Aß and the persistent expression of GluA1-containing AMPARs eventually force the postsynaptic [Ca^2+^] outside of its physiological limits. Prolonged increases of the postsynaptic [Ca^2+^] promote the downregulation of AMPAR expression and prevent GluA1 subunit incorporation into the PSD [71]. It furthermore alters AMPAR kinetics by reducing the channel open probability and the occurrence of subsequent [Ca^2+^] fluctuations [33]. It also enables the mobilization and internalization of AMPARs through clathrin-mediated endocytosis [72]. This process is, among other things, facilitated by endophilin 2, which increases its activity in the presence of Aß [73]. AMPARs are then recycled or degraded which involves ubiquitination and subsequent breakdown by the proteasome. High concentrations of Aß have been shown to promote ubiquitination of AMPARs through activation of the E3 ubiquitin-protein ligase NEDD4 [73, 74]. Aß recruits NEDD4 to the synapse, where it strongly associates with, and promotes the breakdown of, AMPAR subunits. This imbalance in AMPAR physiology eventually initiates a biological cascade of events that involves the pathological regulation of protein and enzyme phosphorylation. Affected proteins include cyclic adenosine monophosphate (cAMP), protein kinase A (PKA), and cAMP response element-binding protein (CREB), which are all essential for inducing gene expression and the maintenance of synaptic plasticity [75–78].

Overall, AMPAR physiology involves a delicate balance between alterations in subunit composition, receptor trafficking, and endo- and exocytosis, all heavily regulated by the postsynaptic [Ca^2+^]. Aß pathology disturbs this balance by promoting the influx of Ca^2+^, ultimately downregulating AMPAR expression and preventing the upregulation of genes essential for synaptic plasticity (Fig. 2).

### Postsynaptic NMDA Receptors

Functional NMDA receptors are heterotetramers that in most cases contain two GluN1 subunits and a combination of GluN2 subunits [79]. The GluN2 subunits contain a glutamate binding site, whereas the GluN1 subunit has high affinity for important co-factors for glutamatergic transmission, such as D-serine and glycine [79]. Sporadically, NMDA receptors include a GluN3 subunit that contains an additional glycine binding site and is characterized by reduced Ca^2+^ permeability [80]. NMDARs are both ligand- and voltage-gated and depend on AMPAR activation for release of the Mg^2+^ block. When activated, NMDARs are highly permeable to Ca^2+^, which acts as an important second messenger and triggers essential downstream pathways.

Reports indicate that Aß directly interacts with NMDAR subunits and cause over-activation of predominantly GluN1- and GluN2-containing NMDARs [81]. Especially the GluN2B NMDAR subunit appears to be prone to Aß pathology [82, 83]. Indeed, direct administration of Aß induced synapse loss and changes in NMDAR-dependent synaptic plasticity only in the absence of GluN2B blockers [84].

Like AMPARs, NMDAR activity is subject to the regulatory processes of receptor trafficking and mobilization. For this, NMDARs depend on membrane scaffolding proteins present in the PSD that ensure attachment to the postsynaptic membrane and actively regulate NMDAR expression and function [85–87]. Aß has been shown to bind directly to some of these proteins in a time- and dose-dependent manner, including to PSD 95 [88], which negatively affected NMDAR activity. Aß also shows high affinity with the cellular prion protein (PrP_c_), an alternative protein present in the PSD. Various studies indicate that interaction of Aß with the PrP_c_ affects NMDAR activity and subsequently reduces synaptic plasticity induction [89, 90]. There is, however, also evidence that activation of the PrP_c_ is not essential for the induction of AD-specific impairments [91, 92].

Aß-PrP_c_ signaling and the activation of NMDARs have been shown to activate downstream pathways important for protein phosphorylation. This includes the activation of Fyn kinase, which has been shown to increasingly phosphorylate the GluN2B subunit in the presence of Aß pathology [93]. The enhanced phosphorylation of NMDAR subunits and related proteins further alters NMDAR trafficking and activity [94].

To summarize, Aß pathology impairs postsynaptic physiology by binding to NMDARs directly, as well as by triggering a pathological cascade of events. The effect of Aß on NMDAR physiology is time- and dose-dependent, where initial exposure to Aß facilitates postsynaptic transmission, but prolonged increases of the postsynaptic [Ca^2+^] ultimately inhibit NMDARs activation and induce postsynaptic depression that involves downregulation of gene expression essential for synaptic plasticity (Fig. 2).

## Astrocytes

**Fig. 3:**
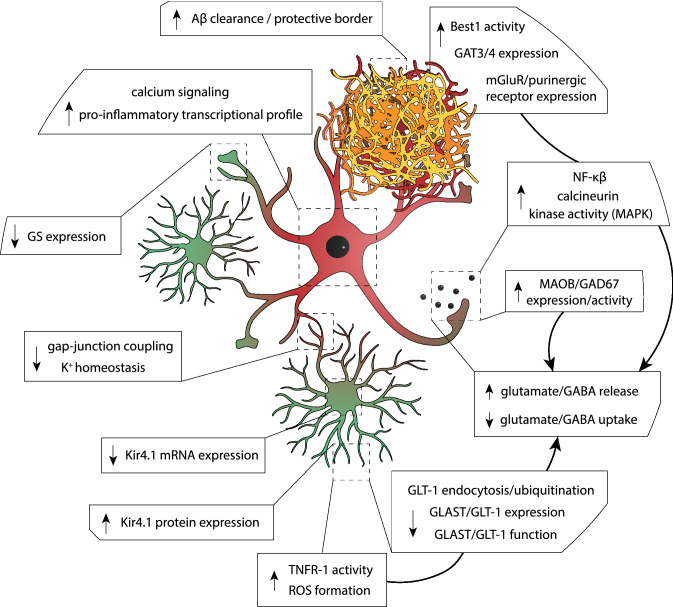
Dysregulation of cellular processes in reactive astrocytes. The figure illustrates a reactive astrocyte displaying differentially regulated processes in response to Aβ pathology. Aβ pathology does not only impact astrocyte function at the single-cell level, but affects the entire astrocyte network. Long-term astrocyte reactivity is associated with a pro-inflammatory transcriptional profile and the decreased expression of neuronal support genes. Functionally, reactive astrocytes display an increase in calcium-wave signaling, which is associated with the increased release of gliotransmitters, including glutamate and GABA. Reactive astrocytes additionally upregulate the expression of several receptors which further stimulates gliotransmitter release. Simultaneously, reactive astrocytes downregulate the expression of glutamate transporters (GLAST/GLT-1), which promotes the presence of glutamate in the synapse. This is further stimulated by the decreased expression of GS. Astrocytes are furthermore important for maintenance of the K^+^ homeostasis and its dysfunction has been implicated in more advanced stages of AD progression, characterized by the decreased expression of Kir4.1 mRNA and impaired gap-junction coupling. In early-stage AD, however, reactive astrocytes ameliorate disease progression by the upregulation of Kir4.1 protein expression near Aβ-plaque enriched areas and protect against Aβ pathology through their active participation in Aβ clearance and the formation of a protective border surrounding the Aβ plaque. Figure was created with the help of BioRender.com

Astrocytes form an intricate part of the central nervous system (CNS) and provide for molecular, cellular and organ homeostasis. Astrocytes are heterogeneous overall and appear in many forms that differ in their morphology, functionality, and physiology. They maintain tight connections with neuronal synapses via perisynaptic processes. It is estimated that a single astrocyte can contact between 20,000 and 120,000 synapses in the rodent brain and up to two million in the human brain [95, 96]. Given the intimate relationship they have with neuronal synapses, astrocytes are essential for neurophysiological signaling. Their function includes the maintenance of tissue ion homeostasis, neurotransmitter recycling, and the regulation of synaptic transmission via the release of gliotransmitters [16, 17, 97]. As such, astrocytes synchronize neuronal activity within brain regions and prevent excitotoxicity of neuronal networks [98–100]. In AD, astrocytes lose their supportive function and change their state towards a pro-inflammatory profile [101]. This shift includes changes in morphology, function, and transcriptional signature (Fig. 3). Reactive astrocytes increase their expression of important intermediate filament proteins that largely consist of glial fibrillary acidic protein (GFAP) and vimentin (VIM) [102, 103]. Functionally, reactive astrocytes display calcium waves that are higher in frequency and longer in duration [104–106] and increase their sensitivity to glutamate through the enhanced expression of AMPARs, but simultaneously reduce the expression of glutamate transporters necessary for neurotransmitter uptake. Generally, chronic astrocyte reactivity is considered detrimental for AD progression. Nevertheless, early astrocyte reactivity has been suggested to be protective against AD pathology due to increased Aβ clearance or the upregulation of proteins important for neurophysiology (Fig. 3) [107–109]. Next, we will discuss the most prominent changes in astrocyte physiology as a consequence of AD pathology.

### Ion Homeostasis

Ion homeostasis is essential for healthy brain functioning. Astrocytes are specifically involved in maintaining K^+^ ion homeostasis and do so through their K^+^ buffering capacity [42, 43]. To effectively carry out their function, the astrocyte membrane is highly permeable to K^+^, causing the astrocyte membrane potential to be very close to the K^+^ equilibrium potential. Neuronal activity increases the extracellular [K^+^], which leads to a difference in the reversal potential of K^+^. A subsequent change in driving force then induces an inward K^+^ current. To efficiently buffer large amounts of K^+^, astrocytes are electrically coupled to neighboring astrocytes [110–113], allowing them to distribute K^+^ towards sites with reduced neuronal activity. Reactive astrocytes change their function in response to Aβ pathology [101], which includes altered gap junction coupling efficiency [114]. Consequently, post-mortem AD patient material and primary astrocyte cultures exposed to Aβ display dysregulated K^+^ and Na^+^ homeostasis [115]. Furthermore, an imbalance in K^+^ homeostasis has been suggested to cause hyperexcitability in mouse models for AD. Astrocytes depend on inward-rectifier K^+^ (Kir) channels for the efficient uptake of extracellular K^+^. Kir channels are a subset of K^+^ channels that favor inward K^+^ currents over outward K^+^ currents. Astrocytes express several Kir channel subtypes, including Kir2.1, Kir4.1, and Kir5.1 [110, 116]. Especially Kir4.1 has been implicated in the aberrant homeostatic function of astrocytes in neurodegenerative diseases. For instance, Kir4.1 function is impaired in epilepsy and Huntington’s disease [117]. In AD, Kir4.1 mRNA expression was found to be downregulated in mice characterized by severe Aβ pathology [118]. Recent evidence, however, suggests that Kir4.1 channel dysfunction is most likely not implicated in the pathology of early AD [107]. Kir4.1 expression was, however, upregulated in astrocytes near Aβ plaques. This implicates that astrocytes in early stages of AD progression try to rectify imbalances in K^+^ homeostasis by upregulating Kir4.1 expression near areas with severe Aβ pathology, and thus are predominantly protective at the early stages of disease progression (Fig. 3) [107].

### Neurotransmitter Recycling

Astrocytes regulate neuronal transmission via the uptake and recycling of neurotransmitters and are especially important for the uptake of glutamate to prevent neuronal network hyperexcitability [119]. Upon presynaptic release, excess glutamate is internalized by astrocyte glutamate transporters, including the l-glutamate/l-aspartate transporter (GLAST) and the glial glutamate transporter-1 (GLT-1) [120]. The uptake of glutamate by astrocytes is essential for healthy neurotransmission and impaired function and expression of GLT-1 and GLAST has been implicated in AD pathology (Fig. 3) [121]. Indeed, reduced glutamate uptake by GLAST and GLT-1 resulted in excitotoxicity in rat organotypic cultures [119]. Glutamate that is taken up by astrocytes is converted into glutamine by the enzyme glutamine synthetase (GS). Glutamine is then shuttled back to the presynaptic neuron [122–124]. Studies using post-mortem AD patient material indicate decreased levels of GLT-1 in the cortex and hippocampus [125, 126]. The reduced expression of GLAST has been implicated by some studies [121, 125], but not all [126, 127]. Similar results were found by studies using astrocyte cultures or animal models for Aβ pathology [128, 129]. For instance, treatment with Aβ reduced glutamate uptake by cultured astrocytes, mainly mediated by reduced GLT-1 function [130–132]. This Aβ-induced reduction in glutamate uptake was associated with reduced expression levels of both GLT-1 and GLAST protein [131]. Studies indicate reduced expression of GLT-1 and GLAST in many brain regions. However, this appears to be regulated in a spatiotemporal-dependent manner. GLAST was only downregulated in adult AßPP23 mice, and not in old AßPP23 mice [133], and APP/PS1 mice showed reduced GLT-1 levels in the cortex, but not in the hippocampus [134]. Nevertheless, the impaired function and expression of GLT-1 and GLAST are clearly implicated in AD pathogenesis. This possibly results from the increased activity of mitogen-activated protein kinases (MAPK) [131], which increasingly phosphorylate GLT-1 and GLAST when exposed to Aß pathology. Alternatively, studies suggest that Aß stimulates GLT-1 internalization and ubiquitination directly [135]. Downregulation of GLT-1 expression also results from adenosine A_2a_ receptors, which are expressed by astrocytes and whose activation has been connected to AD pathology [136, 137]. Alternatively, the presence of reactive oxygen species (ROS) has been implicated in AD pathology as the exposure of the mouse hippocampus and cortex to Aß increased ROS formation [128, 138]. ROS stimulate ubiquitination of proteins, including GLT-1, and subsequent anti-oxidant treatment prevented Aß-induced deficits [135]. Further processes that are involved in the reduced recycling of neurotransmitters include the reduced expression of GS [139], ultimately decreasing neurotransmitter availability at the presynapse. GS expression is, however, differentially regulated per brain region [140], and it is currently unclear whether its reduced expression is a direct effect of Aβ pathology or otherwise results from the reduced availability of astrocyte glutamate.

Astrocytes additionally regulate inhibitory network activity by the uptake of GABA via GABA transporters (GATs). Astrocytes express multiple GATs, including GAT1, GAT2, and GAT3 [141]. Given that GATs function bi-directionally, they are important for both the release and uptake of GABA. Currently, few studies report on the uptake of GABA by astrocytes in AD. There is evidence for the reduced expression of GAT3 in astrocytes obtained from human induced pluripotent stem cells carrying mutations in the APP or splicing enzymes. This resulted in reduced oxidative GABA metabolism mediated by a decrease in the GABA uptake capacity [142]. Accordingly, many studies report on increased GABA concentrations in the AD brain, both in rodents and AD patients [143, 144]. Most studies, however, attribute this effect to increased astrocyte GABA release rather than reduced GABA uptake. Another study reported on increased GABA content in cortical astrocytes of APP/PS1 mice, whereas there were no signs of altered GABA release [145]. They suggest, however, that this might be mediated by the increased synthesis of GABA by astrocytes directly, and additional research is required to find out how changes in GABA uptake specifically relate to AD pathology.

### Gliotransmitter Release

Astrocytes regulate neuronal activity through the release of gliotransmitters, which include GABA, glycine, glutamate, adenosine triphosphate (ATP), and D-serine [146–150]. Whereas glutamate, ATP, and D-serine support excitatory neurotransmission, astrocytes release GABA and glycine to prevent hyperactivity of the neuronal network [144]. Gliotransmitters released by astrocytes act on ionotropic, metabotropic, and purinergic receptors expressed on the pre- and postsynaptic membrane, and as such, astrocytes regulate pre- and postsynaptic activity directly [151].

Hyperactivity of astrocytes in AD pathology is associated with the excessive release of gliotransmitters. Astrocyte gliotransmitter release involves channel-mediated release [152] and calcium-mediated exocytosis [153, 154]. Given that reactive astrocytes display enhanced calcium-wave activity [104, 105], astrocyte glutamate and D-serine release are characteristically upregulated in AD pathogenesis [155, 156].

Activation of purinergic receptors and ionotropic and metabotropic glutamate receptors (mGluRs) promotes astrocyte glutamate and D-serine release [148, 149, 152, 157]. Studies report that Aβ affects astrocyte receptor expression and function directly. For instance, Aβ administration increased the expression of P2X purinergic receptors [158], and mGluR subtypes [159, 160], as well as the activity of α7-nAChRs [155]. Aβ further induced overexpression of mGluRs by astrocytes via downstream activation of calcineurin and the protein complex NF-κB [160]. This increase in receptor expression and activity has been implicated in the excessive release of astrocyte glutamate, predominantly via the facilitation of calcium-mediated exocytosis [30, 31, 155]. Indeed, administration of Aβ induced a Ca^2+^-dependent increase in serum glutamate levels in cultured astrocytes [156]. Moreover, astrocytes released glutamate after activation of the tumor necrosis factor receptor 1 by tumor necrosis factor α (TNF-α) in a Ca^2+^-dependent manner [161], which was found to be altered in PDAPP mice [162]. This increase in Ca^2+^-dependent glutamate release is possibly mediated by downstream pathways that result in enhanced kinase activity, including the activity of MAPK [163]. Moreover, the increased expression of especially astrocyte purinergic receptors coincides with synaptic failure, thereby linking Aβ pathology to reduced synaptic transmission via the altered function of astrocytes [158].

To maintain physiological network activity, astrocytes release GABA through reversal of GATs and channel-mediated release, including the activation of Bestrophin-1 (Best1) [164–166]. Astrocyte GABA release activates neuronal ionotropic GABA_a_ and metabotropic GABA_b_ receptors [147]. As such, astrocytes contribute to the tonic inhibition of neuronal networks upon the excessive release of glutamate [167], further confirming their role in network synchronization. GABAergic circuits are affected by AD pathology, as suggested by studies showing increased GABA concentrations in the hippocampus of AD patients and AD mouse models, especially near Aβ plaques [143, 144]. Experiments suggest that reactive astrocytes increase their expression of GAT3 and GAT4 [144], and use those to promote GABA release [165]. Indeed, the increased release of GABA by reactive astrocytes caused tonic inhibition in the hippocampus, only in the absence of GAT3 and GAT4 inhibitors [144]. Hyperactivity of Best1 is also implicated in the increased release of GABA by reactive astrocytes, as Best1 short-hairpin RNA prevented enhanced GABA expression in cultured astrocytes [143]. Astrocytes generate GABA directly via the glutamic-acid-decarboxylase (GAD) and glial-monoamine-oxidase B (MAOB) pathways [168, 169]. Recent evidence indicates that astrocytes primarily depend on the MAOB pathway [169]. Its activity was found to be upregulated when exposed to Aβ pathology in AD model mice and post-mortem AD patient material [143, 170]. Moreover, glutamic acid decarboxylase 67 (GAD67) was found to be significantly increased in reactive astrocytes in the dentate gyrus of 5xFAD mice [144]. Overall, the increased release of GABA by reactive astrocytes reduced the presynaptic release probability and excitability of neurons in the dentate gyrus [143]. This ultimately resulted in the impaired induction of synaptic plasticity and cognitive deficits [143, 144].

To summarize, whereas astrocytes generally ensure physiological activity of the neuronal network, Aβ pathology induces excessive release of excitatory and inhibitory gliotransmitters, including glutamate and GABA (Fig. 3). Their release might be differentially regulated, as hippocampal GABA content was found to be upregulated mainly at later stages of AD progression near Aβ plaques [170, 171]. On the contrary, hyperexcitability of neuronal networks is typically present in early stages of Aβ pathology [45]. This implicates that the regulation of neuronal activity by astrocytes in AD is affected in a spatiotemporal-dependent manner.

## Microglia

Microglia are the brain’s primary immune cells and are the first to act in case of neuronal injury. Microglia respond to a variety of signals that lead to polarization into distinct phenotypes, both pro-inflammatory and anti-inflammatory [172]. This activation is essential for a proper induction and subsequent resolution of the brain’s immune response. Furthermore, microglia act as the primary regulators of neuronal plasticity during development and adulthood [21, 173–175]. For this, they regulate the elimination of inactive synaptic connections and maintain functional synapses by a process often referred to as “synaptic stripping” or “pruning” [20, 176, 177]. Neurons form an excess of synapses during development and microglia eliminate weak or unnecessary synapses based on neuronal activity [178]. Furthermore, microglia are regarded as important mediators of neuronal plasticity by the release of cytokines and the expression of enzymes [21, 179, 180]. Throughout these processes, the microglial phenotype is highly dynamic, and mature microglia contain a vast number of receptors to quickly respond to changes in their environment [181, 182]. This response includes microglial activation, which is accompanied by transcriptional and phenotypic changes that are essential for a proper response to any disturbance of brain homeostasis [182–184].

### Microglia Phenotypes

**Fig. 4:**
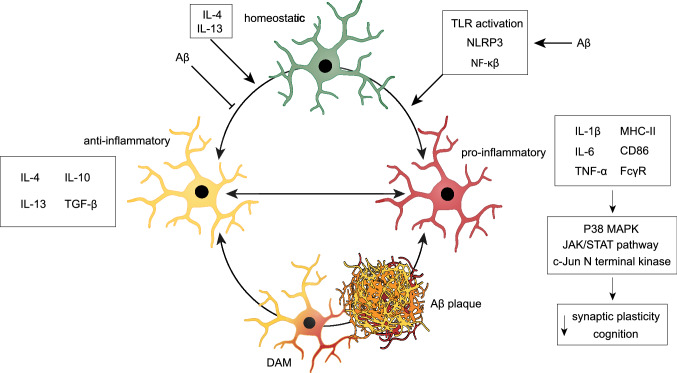
Microglia are highly dynamic throughout various physiological states. Homeostatic microglia monitor the ingress of pathogens and interact directly with neurons, which is essential for regulating synaptic plasticity. Threats to the structural and functional integrity of the CNS may lead to microglial polarization towards various activated states. The presence of anti-inflammatory cytokines in the microenvironment pushes microglia towards an anti-inflammatory state, which is essential in the resolution of the immune response. In AD, microglia initially participate in Aβ clearance through their phagocytotic capacity. However, the continued exposure to soluble and oligomeric Aβ induces a pro-inflammatory microglial response through TLR, NLRP3 inflammasome, and NF-κB signaling. These microglia release pro-inflammatory cytokines that affect the capability of neurons to induce synaptic plasticity via the activation of downstream pathways involving P38 MAPK and c-Jun N terminal kinase signaling and activation of the JAK/STAT pathway. Moreover, microglia lose their potential to mount an anti-inflammatory response as a result from Aβ accumulation. DAM are microglia specifically associated with Aβ-plaque pathology in AD. DAM are neuroprotective in the initial stages of AD pathology but due to chronic stimulation become increasingly pro-inflammatory with disease progression. Figure was created with the help of BioRender.com

Until recently, the microglia phenotype was subdivided according to the M1/M2 classification. Recent insights, however, have led to the understanding that the microglia phenotype includes a spectrum of states between which microglia can freely transition based on the signals present in the microenvironment (Fig. 4) [185]. Under physiological circumstances, microglia are characterized by a branched morphology and their protrusions continuously undergo cycles of formation and withdrawal to scavenge/scan the extracellular environment [182]. This process plays a key role in monitoring the ingress of pathogens and detection of neuronal damage [186]. Moreover, microglia interact directly with neurons, preferentially contacting neurons with high levels of activity [187], which is essential for regulating synaptic plasticity. Microglia maintain their ramified morphology through homeostatic neuronal and astrocyte signaling [188]. Threats to the structural and functional integrity of the CNS may, however, lead to microglial activation towards an anti-inflammatory or pro-inflammatory state [172]. These states are accompanied by changes in appearance, including enlargement of the soma and reduced ramification.

Microglia become polarized towards a pro-inflammatory state as a response to pathogens, trauma, or ischemia [189–192]. Their main function is to mount an adaptive immune response. Accordingly, a pro-inflammatory response by microglia is characterized by an increased antigen-presenting activity through upregulation of the major histocompatibility complex II, CD86, and Fc-γ receptors, allowing for improved crosstalk between immune cells [193–196]. Downstream signaling pathways of these receptors lead to the release of pro-inflammatory signaling molecules, such as interleukin 6 (IL-6), interleukin 1-bèta (IL-1ß), and TNF-α (Fig. 4) [195, 197, 198].

Alternatively, the presence of anti-inflammatory cytokines in the microenvironment induces microglia differentiation towards an anti-inflammatory state, which is essential for dampening of the pro-inflammatory response [199, 200]. These cytokines include interleukin 4 (IL-4), and interleukin 13 (IL-13), secreted by immune cells at the end of an inflammatory response [201, 202]. In response, microglia secrete a range of anti-inflammatory factors, including IL-4, IL-13, interleukin-10 (IL-10), and transforming growth factor beta (Fig. 4) [202, 203]. Anti-inflammatory cytokine signaling also induces the upregulation of scavenger receptors and enhancement of neurotrophic factor release [200, 204]. These promote debris clearance and resolution of inflammation, respectively. The anti-inflammatory response of microglia is implicated in neuronal protection and repair. Indeed, inhibiting the anti-inflammatory response by microglia worsened pathology after neuronal damage induced by stroke [205].

### Microglia in AD

In AD, microglia are tightly associated with Aß plaques and their activation may play a complex but dual role [206]. For instance, microglia are known for the uptake and degradation of Aß, thereby initially counteracting AD pathology [206, 207]. Prolonged exposure to Aß, however, pushes microglia towards a pro-inflammatory phenotype, which plays a key role in neuro-inflammation and neurodegeneration by secreting a range of pro-inflammatory cytokines, indicated by increased levels of IL-1ß and TNF-α (Fig. 4) [208, 209].

Underlying the pro-inflammatory response of microglia is a broad intracellular signaling cascade, involving both toll-like receptors (TLRs) and the nod-like receptor protein 3 (NLRP3) inflammasome [210, 211]. Microglia express a range of TLRs that are essential in recognizing harmful stimuli and in the induction of the innate immune response [212, 213]. In AD, TLRs fulfil a dual role. Whereas TLR2 and TLR4 are important for the phagocytosis of Aß by microglia [214], loss-of-function-mutations in the TLR4 gene result in significantly decreased microglial activation and release of pro-inflammatory cytokines [211]. Moreover, deficiency for TLR4 proved to be sufficiently protective against microglia activation, neuro-inflammation, and subsequent memory impairments in mice exposed to Aß [215]. The transcription factor NF-κB acts as a downstream effector of TLR4 and its activation results in the expression of pro-inflammatory cytokines, including TNF-α, IL-6 and IL-1 [216]. NF-κB additionally triggers transcription and activation of the NLRP3 inflammasome, which promotes the formation and secretion of IL-1ß [210, 217, 218]. Aß also stimulates the NLRP3 inflammasome directly, which further promotes IL-1ß production and microglia reactivity [210, 219, 220]. A similar mechanism applies to human AD pathology, as hippocampal lysates from AD patients with mild cognitive impairments showed elevated caspase 1 concentrations, which is an important component of the NLRP3 inflammasome [210]. Additional pathways implicated in the Aß-induced activation of the NLRP3 inflammasome are cathepsin activation [221] and Ca^2+^-mediated activation of the calcium-sensing receptor [222]. The importance of NLRP3 inflammasome activation in microglia reactivity becomes apparent from experiments indicating that NLRP3 deficiency successfully protects against microglia activation, decreases Aß accumulation, and prevents spatial memory loss [210, 219].

While the secretion of pro-inflammatory cytokines is initially important for driving microglial activation and Aß phagocytosis [223–225], excessive release of pro-inflammatory cytokines is linked to neurotoxicity and reduced synaptic plasticity [175, 226, 227]. For example, overexpression of IL-1ß led to a significant decrease in long-term contextual and spatial memory in mice [228], whereas inhibition of pro-inflammatory signaling in AD mouse models significantly decreased cognitive deficits [175, 229]. Moreover, exposure to TNF-α decreased spatial memory performance in mice [230] and reduced hippocampal synaptic plasticity [231]. Studies indicate that both IL-1ß and TNF-α activate p38 MAPK [231, 232], which subsequently stimulates NMDAR phosphorylation [233] and glutamate receptor-dependent long-term depression [234]. p38 MAPK additionally inhibits brain-derived neurotrophic factor, an important positive modulator of synaptic plasticity [235, 236]. Indeed, selective inhibition of p38 MAPK rescued synaptic plasticity in the cortex of AD model mice [237]. TNF-α has further been implicated in impaired synaptic plasticity via interaction with the c-Jun N-terminal kinase [238], whose activation has been implicated in AD pathology and inhibition leads to significant improvements in cognitive performance [239]. In addition to IL-1ß and TNF-α, exposure to IL-6 reduced synaptic plasticity induction *in-vitro* in hippocampal neurons [240, 241] and downregulated synaptic protein expression, including AMPAR subunits [242]. Chronic exposure to IL-6 further induced neuronal circuitry imbalance and deficits in learning and memory in adult mice [243], possibly through activation of the JAK/STAT3 pathway [244, 245].

The constant release of pro-inflammatory cytokines results in a vicious cycle between neuronal tissue damage and subsequent inflammation. Since inflammation and increased levels of pro-inflammatory cytokines are an intrinsic part of AD, pro-inflammatory microglia are proposed to play an important role in AD pathology. The increased neuro-inflammation in AD suggests that the anti-inflammatory response of microglia and corresponding release of anti-inflammatory factors are downregulated. Indeed, AD progression is associated with a microglial switch towards an increasingly pro-inflammatory phenotype in AD model mice [246]. Furthermore, with age, microglia become less responsive to signals that resolve the pro-inflammatory response [247], which further stimulates neuro-inflammation (Fig. 4).

Recently, the interpretation of the balancing process between pro-inflammatory and anti-inflammatory microglia became increasingly complex with the discovery of a microglial signature exclusively present in neurodegenerative diseases, including AD. These “disease-associated microglia” (DAM) were first discovered in a mouse model of AD in proximity to Aß plaques [248]. Later, DAM were detected in models for tau pathology and in post-mortem brain tissue of AD patients [249]. DAM display a unique transcriptional profile that includes the downregulation of homeostatic microglial genes and the upregulation of genes involved in phagocytosis and lipid metabolism [248]. Their close proximity to Aß plaques and their increased phagocytotic capacity implicates a protective role in AD (Fig. 4), further endorsed by the increased expression of triggering receptor expressed on myeloid cells 2 (TREM2), a receptor known to facilitate Aß degradation [250]. Recent studies indicate, however, that DAM also include pro-inflammatory subtypes [251]. As such, it has been proposed that DAM are neuroprotective in the initial stages of AD progression by phagocytosing Aß, which due to chronic stimulation transitions into a pro-inflammatory state. As such, the AD microenvironment pushes microglia from a homeostatic to an increasingly pro-inflammatory state, while at the same time inhibiting the anti-inflammatory response. This shift in microglia signature has detrimental consequences for tissue homeostasis and neurophysiology, ultimately leading to cognitive decline.

### Microglia and Synapse Loss

Synapse loss is a major characteristic of early-stage AD [25]. The total number of synapses decreases significantly in AD patients and this decrease positively correlates to cognitive decline [252]. Underlying this reduction is a process called synaptic pruning, which involves the engulfment and removal of synapses by microglia. The elimination of synapses is likely based on their activity. Indeed, microglia eliminate weak or unnecessary synapses, based on neuronal activity in the visual cortex [253]. Furthermore, it was confirmed that synapse elimination is necessary for the development of mature brain circuitry in the hippocampus [20]. The pro-inflammatory microglia response has been implicated in excessive synaptic pruning in many neurodegenerative diseases, including AD [19, 254]. Microglia regulate synaptic pruning via several pathways, including activation of the CX3C chemokine receptor 1 (CX3CR1) and the complement system. Fractalkine (a.k.a. CX3CL1) functions as a ligand for the CX3CR1 and is a chemokine expressed by neurons in a membrane-anchored and soluble form. Fractalkine acts as a synaptic chemoattractant and induces microglial synapse engulfment through binding to its microglial receptor [255, 256]. Accordingly, fractalkine knock-out mice show defective synaptic pruning in the developing hippocampus [20]. In AD, the dysregulation of synaptic pruning is also regulated by the complement system and Aß is able to bind directly to complement system components [21, 257]. Moreover, complement factors were found upregulated in the cerebrospinal fluid of AD patients with mild cognitive impairments [258]. The complement system allows for the opsonization and subsequent phagocytosis of pathogens and cellular debris via complement factor signaling. Synaptic pruning is induced by the synaptic expression of complement components 1q (C1q) and 3 (C3). The C3 complement receptor (CR3) is specific for microglia and binding of processed C3 leads to phagocytosis of the synapse in an activity-dependent manner [255, 256]. Evidence indicates that processed C3 preferentially co-localizes with weaker synapses during development of the visual system [178]. Furthermore, it was determined that disruption of the CR3/C3 pathway leads to an increased synaptic density and increased excitatory neurotransmission. In AD, the CR3/C3 pathway is most likely over-activated, as C3 deficiency appeared to protect against hippocampal synapse loss in an AD mouse model [254]. Moreover, C1q and C3 were found upregulated preceding synapse loss in a mouse model for AD and mice deficient for C1q, C3, or C3R completely rescued the reduction in synapse density [19]. The involvement of microglia in synaptic pruning was further confirmed by a recent study that indicates increased C3R-dependent phagocytosis in microglia-neuron co-cultures upon the administration of Aß, involving a process called desialylation [259]. Together, this suggests that synaptic pruning as a result of pro-inflammatory microglia activation plays an important role in AD pathology.

## Conclusion

The literature discussed here indicates that AD is a multimodal neurodegenerative disease that includes the pathophysiology of neurons, astrocytes, and microglia. Their functions integrate at the synapse, which physiology is essential for healthy brain functioning and cognitive performance. In AD, the excessive accumulation of Aß pushes the synapse away from its physiological equilibrium towards a pathophysiological state. This switch is accompanied by similar changes in astrocyte and microglia function. Astrocytes and microglia are initially protective in AD pathology and try to rectify abnormal synaptic transmission by participating in Aß clearance and the compensatory expression of functional proteins. Nevertheless, chronic activation of astrocytes and microglia works aversive and provides an additional trigger for aberrant synaptic transmission. Reactive astrocytes lose their supportive function and microglia transition towards a pro-inflammatory phenotype. Cytokines released during this inflammatory response act on neurons, astrocytes, and microglia directly, providing an important cross-link between cells of different origins in AD pathology. Studies increasingly focused on the interplay between different cell types using novel approaches, such as spatiotemporal transcriptomics and the use of transgenic (mouse) models conditionally expressing cell-type specific mutations [260–262]. Yet, the high similarity between affected mechanisms in different cell types and the current technical possibilities make it challenging to unravel functional dynamic changes at the synapse with a high spatiotemporal resolution. Furthermore, the tight interaction between components of the quad-partite synapse and the highly dynamic processes that are at play make it difficult to determine the timescale at which changes occur throughout AD pathogenesis. The high diversity of mouse models used in AD studies contributes to this and makes it difficult to interpret the impact of new findings on a larger scale. Still, advances have been made in recent years, with new insights into how astrocytes and microglia function as direct modulators of synaptic plasticity [180, 263]. This shows that the way in which astrocytes and microglia are implicated in synaptic plasticity is even more complex than previously anticipated and this raises the question of how similar mechanisms relate to AD pathology. Overall, AD etiology involves a complex interplay between (epi)genetic changes and environmental risk factors, that ultimately leads to changes at the microcircuit level. Future studies are warranted to unravel these functional changes in more detail using new experimental approaches that allow manipulation of neuron–glia interactions with high spatiotemporal resolution, with the hope of developing novel treatments for AD.

## Data Availability

Enquiries about data availability should be directed to the authors.
